# From State, Pathway, to Niche: The Ternary Network of Breast Cancer Stem-like Cells Driving Tumor Progression and Combination Therapy Prospects

**DOI:** 10.3390/biom16050645

**Published:** 2026-04-26

**Authors:** Sitong Man, Lei Zhang, Bo Chen

**Affiliations:** Department of Breast Surgery, The First Hospital of China Medical University, Shenyang 110001, China; mansitong@cmu.edu.cn (S.M.); zhangl2005@cmu.edu.cn (L.Z.)

**Keywords:** breast cancer stem-like cells, immune microenvironment, therapeutic resistance, immune evasion, targeted therapy, precision medicine, artificial intelligence

## Abstract

Breast cancer stem-like cells (bCSCs) fundamentally represent a highly dynamic “immune-adaptive functional state” rather than a fixed cellular lineage, serving as the core engine driving tumor recurrence, metastasis, and therapeutic resistance. Despite rapid advances, the heterogeneity of bCSC states and their intricate interactions with the immune microenvironment lack systematic integration. This review centers on the dynamic evolution and niche adaptation of bCSCs. First, we systematically dissect the multilayered regulatory network maintaining stemness, encompassing core transcription factors, epigenetic–metabolic coupling, and the synergistic mechanisms of critical signaling pathways such as Wnt and Notch. Second, we propose a trinary “stemness–immune–spatial” feedback model, elucidating how bCSCs achieve active immune evasion by downregulating antigen presentation, secreting immunosuppressive factors, and embedding within perivascular “immune-cold niches.” Finally, leveraging a multi-omics integration perspective, we reconstruct precision intervention strategies, exploring the synergistic potential of targeting stemness pathways in conjunction with immunotherapies like PD-1/PD-L1 blockade and STING agonists. Furthermore, we highlight the pivotal role of integrating organoids, PDX models, and AI-assisted decision systems in overcoming heterogeneity and enabling personalized treatment. By establishing a closed-loop framework spanning mechanistic insight to spatially precise intervention, this review aims to provide novel theoretical foundations and translational pathways to surmount the bottleneck of therapeutic resistance in breast cancer.

## 1. Introduction

Breast cancer (BC) is one of the most prevalent and deadly malignancies among women worldwide, characterized by pronounced clinical heterogeneity, encompassing diverse molecular subtypes, histopathological features, and therapeutic response patterns [1,2]. Although recent advances in molecular subtyping, targeted therapy, and immunotherapy have shown promise, the persistent challenges of long-term recurrence, metastasis, and therapeutic resistance remain unresolved. These issues suggest the involvement of an underlying “root” cellular population driving disease progression [3,4]. An increasing body of evidence has identified a subset of BC cells with stem cell-like properties—termed cancer stem-like cells or cancer stem cells (CSCs)—as potential key players in tumor evolution and immune evasion [5,6,7].

**Figure 1:**
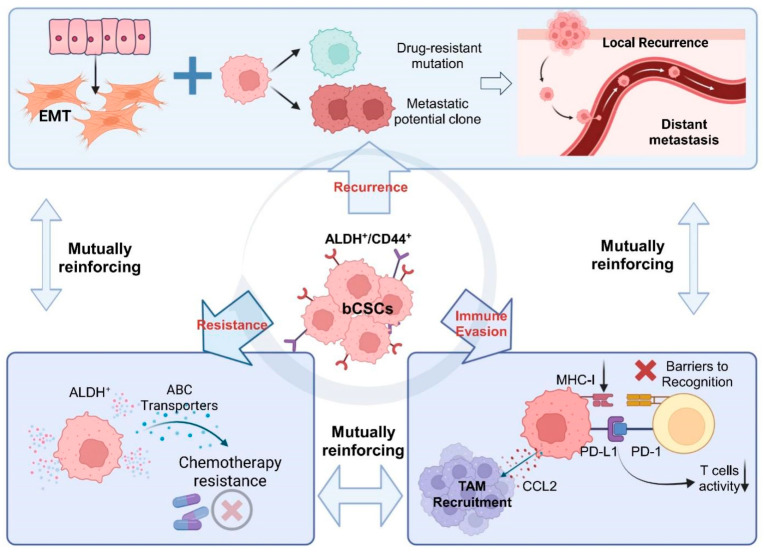
Overview of the pivotal role of bCSCs in driving breast cancer recurrence and immune evasion. Note: Breast cancer stem-like cells (bCSCs, typically ALDH^+^/CD44^+^) are positioned at the core, driving three major functional axes in a radial structure: ① promoting local recurrence and distant metastasis via mechanisms such as epithelial–mesenchymal transition (EMT) and clonal selection; ② mediating chemotherapy resistance through ALDH activity and ABC transporter-mediated drug efflux; ③ facilitating immune evasion by downregulating MHC-I, upregulating PD-L1, and recruiting tumor-associated macrophages (TAMs). These three functional modules are mutually reinforcing: drug-resistant subclones often exhibit enhanced EMT and metastatic potential, while the immune-evasive microenvironment (e.g., factors secreted by TAMs) reciprocally promotes stemness maintenance and chemotherapy resistance. Together, they form a dynamic and reversible stemness state network, highlighting the central role of bCSCs in tumor progression and immune regulation. Abbreviations: EMT: Epithelial–Mesenchymal Transition; ALDH: Aldehyde Dehydrogenase; CD44: Cluster of Differentiation 44; bCSCs: Breast Cancer Stem Cells; ABC Transporter: ATP-Binding Cassette Transporters; MHC-I: Major Histocompatibility Complex Class I; PD-L1: Programmed Death-Ligand 1; PD-1: Programmed Cell Death Protein 1; TAM: Tumor-Associated Macrophages; CCL2: C-C Motif Chemokine Ligand 2. (Figure created by BioRender).

The existence of breast cancer stem-like cells (bCSCs) has been validated in multiple animal models and clinical specimens, and they are highly enriched in aggressive subtypes such as triple-negative breast cancer (TNBC) [8]. These cells are characterized by sustained self-renewal capacity, pronounced therapeutic resistance, high metastatic potential, and potent immunosuppressive properties. Consequently, bCSCs are widely regarded as key cellular drivers underlying tumor recurrence and metastatic dissemination (Figure 1) [5,7,9]. Several patient-derived xenograft (PDX) model studies have indicated that bCSCs with the aldehyde dehydrogenase-positive (ALDH^+^)/cluster of differentiation 44-positive (CD44^+^) phenotype are commonly associated with enhanced chemoresistance and metastatic capacity, suggesting that this subpopulation may represent a highly aggressive “functional core stemness cluster” [7,10]. Furthermore, stem-like cells remodel the immune microenvironment by secreting factors such as interleukin-6 (IL-6) and C-C motif chemokine ligand 5 (CCL5), thereby suppressing CD8^+^ T-cell activity and activating regulatory T cells (Tregs) and tumor-associated macrophages (TAMs). These findings indicate that bCSCs modulate the immune barrier by dynamically regulating stemness states, positioning them as critical mediators of immune evasion in BC [11,12]. Therefore, identifying and elucidating the key regulatory mechanisms of stem-like cells holds substantial clinical value for improving long-term survival outcomes in BC patients.

Although bCSCs are often categorized under the umbrella of CSCs, recent studies increasingly regard them as a dynamically plastic functional state rather than a fixed cellular lineage [5,9]. bCSCs can be induced from non-stem tumor cells under specific microenvironmental stimuli, such as hypoxia or immune pressure, exhibiting both reversibility and heterogeneity in their state [6,13]. Accordingly, this review alternates between the terms “stem-like cells” and “stemness state” to underscore their lineage-independent functional nature.

While traditional bulk transcriptomic approaches (e.g., bulk RNA sequencing [bulk RNA-seq]) have provided pathway-level insights, they are inherently limited in capturing the cellular-level heterogeneity underlying stemness lineages and immune interactions. With the maturation of single-cell RNA sequencing (scRNA-seq) and its integrative analysis with bulk data, researchers have been able to systematically delineate the lineage trajectories, functional transitions, and microenvironmental embedding of stemness states [14,15,16]. For instance, several studies combining scRNA-seq and spatial transcriptomics have demonstrated that stem-like cells can cooperate with immunosuppressive cells—such as TAMs—via chemokine axes (e.g., CXCL-related pathways) to shape immune-exclusionary niches, thereby offering novel avenues for stemness–immune co-targeting strategies [15,17,18].

In addition, with the continued advancement of multi-omics integration technologies—such as scRNA-seq, bulk RNA-seq, assay for transposase-accessible chromatin using sequencing [ATAC-seq], and spatial omics—the lineage plasticity, metabolic dependencies, and epigenetic regulatory pathways of stem-like cells have been gradually incorporated into a multidimensional mechanistic framework [16,19]. Studies integrating scRNA-seq data with ATAC-seq have shown that the homeostatic maintenance of bCSCs relies on the coordinated regulation of multiple signaling and metabolic pathways, including the crosstalk between networks such as Wnt signaling and cholesterol metabolism. Among these, transcriptional regulators such as the FOXC family have been identified as potential hubs within this interactive axis [20].

Collectively, bCSCs function as the central “nodal units” driving breast cancer heterogeneity, recurrence, and immune evasion, increasingly positioning them as potential targets for precision therapy. However, current reviews predominantly center on isolated signaling pathways or therapeutic modalities, lacking a systematic integration of the “Lineage–Immune–Spatial” trinary interaction mechanisms governing the stemness state. This limitation hinders the development of depth-oriented targeting strategies. Accordingly, this review addresses three critical questions that serve as its structural framework: first, the identification characteristics and multi-pathway regulatory mechanisms of the bCSC state; second, the reciprocal reshaping network between stemness and the immune microenvironment, along with its spatial embedding features; and third, the reconstruction of ecological architecture and precision intervention strategies based on multi-omics integration. Furthermore, we emphasize that the stemness state represents a highly dynamic, immune-adaptive functional phenotype. We introduce a novel precision intervention pathway integrating organoids, PDX models, and AI-assisted decision-making, aiming to propel the translational leap from mechanistic insight to clinical application.

## 2. State Identification and Multidimensional Regulatory Mechanisms of bCSCs

### 2.1. Identification Systems and Marker Profiles of bCSCs

bCSCs represent a functional subpopulation with tumor-initiating potential, self-renewal capacity, and differentiation plasticity, and are considered the root cellular drivers of BC recurrence, metastasis, and therapeutic resistance [21,22,23]. The strategies for bCSC identification have evolved from traditional functional assays to phenotype-based definitions and, more recently, to integrated systems combining multi-omics profiling and AI-based predictive scoring [6,7,24].

Initially, the identification of bCSCs relied primarily on functional assays such as limiting dilution transplantation and sphere-forming (mammosphere) assays. These approaches directly validated their tumor-initiating capacity but were technically demanding, low-throughput, and unsuitable for large-scale clinical analysis [25]. Subsequently, researchers established rapid identification systems based on specific phenotypic markers [26,27,28]. For example, combinations such as CD44^+^/CD24^−^, ALDH^+^, and EpCAM^+^/CD49f^+^ have been used to identify bCSC-enriched populations across different BC subtypes (Table 1) [29,30]. Notably, ALDH enzymatic activity has demonstrated stability in TNBC and has been closely associated with chemoresistance and immune-cold niches [7,27,28].

With the advent of the multi-omics era, single-cell technologies such as scRNA-seq and cellular indexing of transcriptomes and epitopes by sequencing (CITE-seq) have enabled high-resolution, dual-dimensional profiling of transcriptomic and proteomic landscapes. When combined with lineage-trajectory modeling algorithms such as Monocle3 and CytoTRACE, these tools have enabled mapping of bCSC developmental trajectories and functional heterogeneity [34,35]. Spatial transcriptomics platforms (e.g., 10× Visium, GeoMx DSP, NanoString Technologies, Seattle, WA, USA). have further revealed the spatial embedding patterns of bCSCs within the tissue microenvironment, where they are frequently localized in hypoxic niches, perivascular regions, or areas encapsulated by cancer-associated fibroblasts (CAFs), highlighting their niche dependency [36,37,38].

Additionally, AI-assisted systems have been increasingly applied to bCSC identification. Stemness scoring systems trained on expression profiles, such as mRNAsi, have been used to indirectly estimate bCSC enrichment from large-cohort bulk RNA-seq data, enabling immune–stemness risk stratification and quantification across patient subgroups [39].

**Figure 2:**
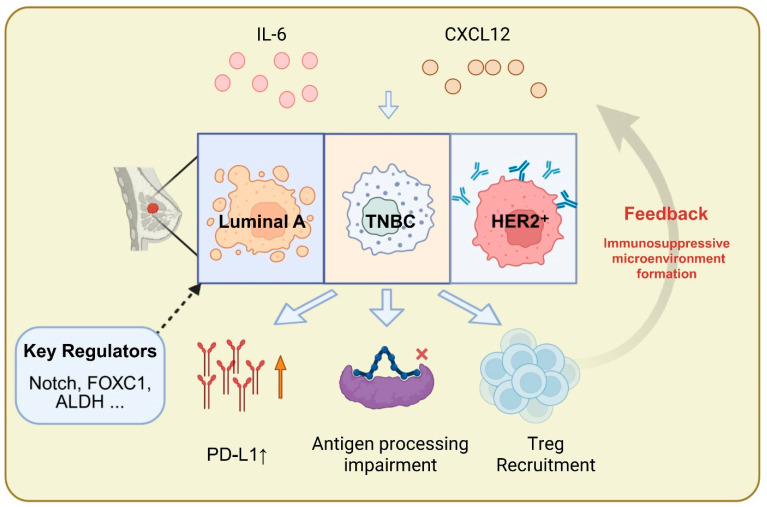
Microenvironment–subtype–phenotype triaxial regulatory model of bCSC phenotypes across BC subtypes. Note: Distinct BC subtypes exhibit subtype-specific bCSC phenotypes under the influence of microenvironmental cues. Luminal A, TNBC, and HER2^+^ subtypes are respectively associated with immune evasion features such as PD-L1 upregulation, impaired antigen processing, and Treg recruitment, modulated by key regulatory factors including Notch, FOXC1, and ALDH. A feedback loop among microenvironmental signals, subtype characteristics, and bCSC immune phenotypes contributes to the establishment of an immunosuppressive microenvironment, suggesting that the immunological heterogeneity of bCSCs across subtypes holds promising implications for targeted intervention. Abbreviations: IL-6: Interleukin-6; CXCL12: C-X-C Motif Chemokine Ligand 12; TNBC: Triple-Negative Breast Cancer; HER2^+^: Human Epidermal Growth Factor Receptor 2-Positive; PD-L1: Programmed Death-Ligand 1; Treg: Regulatory T-Cell; ALDH: Aldehyde Dehydrogenase. (Figure created by BioRender).

Importantly, bCSC phenotypes differ significantly among BC subtypes. These differences may be driven by variations in the activation of stemness-associated transcription factors (TFs) (e.g., SRY-box transcription factor 2 [SOX2], forkhead box C1 [FOXC1]), divergent immune pathway states, and distinct expression profiles of signaling axes such as C-X-C motif chemokine ligand 12 (CXCL12) and IL-6 within the microenvironment [5,35,40]. Some studies have found that bCSCs in human epidermal growth factor receptor 2-positive (HER2^+^) subtypes preferentially express Notch-related factors, whereas those in TNBC are enriched for ALDH^+^ bCSCs associated with immune-cold niches. These findings suggest the presence of a tripartite regulatory relationship among microenvironment, subtype, and phenotype, warranting further systematic validation (Figure 2) [37,41]. To facilitate a clearer understanding of the distinctive properties of bCSCs, Table 2 provides a systematic comparison between bCSCs and non-stem tumor cells (non-bCSCs), highlighting key differences in self-renewal capacity, metabolic programs, and immunological features.

In summary, there is currently no unified marker system for bCSC identification, and significant phenotypic variability exists across different BC subtypes and models [21,40]. Future identification strategies are expected to evolve from marker-based definitions toward integrative models that combine multi-omics, spatial transcriptomics, and AI prediction, thereby improving the precision of stemness assessment and enhancing the feasibility of clinical application [42].

### 2.2. Transcriptional–Metabolic–Epigenetic Coupling in Stemness Lineage Regulation

The maintenance of stemness in bCSCs relies on the tight integration of transcriptional control, epigenetic remodeling, and metabolic reprogramming. Core transcription factors, including MYC, SOX2, and FOXC1, form central regulatory hubs that drive stemness programs, while their activity is finely tuned by chromatin-modifying factors such as EZH2 through dynamic epigenetic regulation [43] (Table 3).

**Figure 3:**
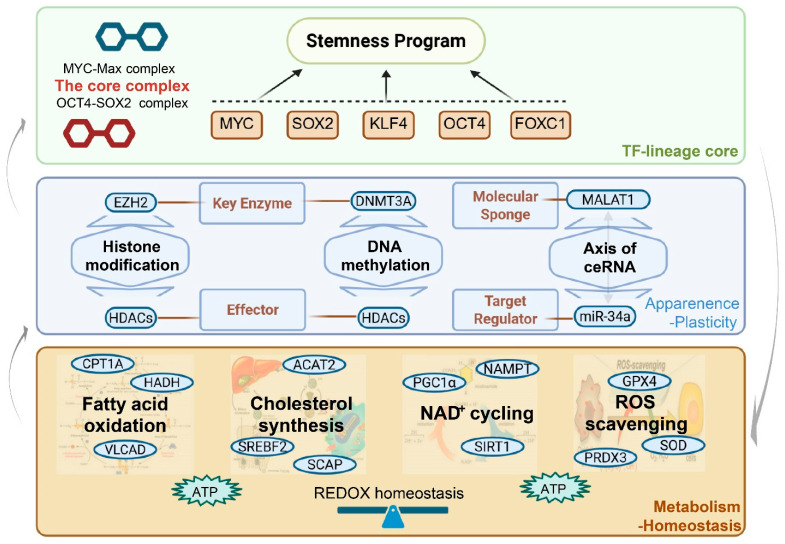
Regulatory network of the transcription–metabolism–epigenetics tri-axis in BC stemness maintenance. Note: The maintenance of stemness in bCSCs is governed by a multilayered regulatory network. At the metabolic level, the lower tier ensures energy supply and redox homeostasis through FAO, cholesterol biosynthesis, nicotinamide adenine dinucleotide (NAD^+^) cycling, and ROS scavenging. The intermediate epigenetic module includes histone modifications (e.g., EZH2, histone deacetylases [HDACs]), DNA methylation (e.g., DNA methyltransferase 3 alpha [DNMT3A]), and competing endogenous RNA (ceRNA) axes, such as MALAT1-miR-34a, that confer plasticity to the stem-like state. At the upper level, a core set of TFs—including SOX2, KLF4, octamer-binding transcription factor 4 (OCT4), MYC, and FOXC1—directly activate the stemness gene program. Bidirectional feedback among metabolic, epigenetic, and transcriptional layers forms a dynamic closed-loop network that underpins the coexistence of homeostasis and plasticity in bCSC states. Abbreviations: MYC: MYC Proto-Oncogene; OCT4: Octamer-Binding Transcription Factor 4; SOX2: SRY-Box Transcription Factor 2; KLF4: Krüppel-Like Factor 4; FOXC1: Forkhead Box C1; TF: Transcription Factor; EZH2: Enhancer of Zeste Homolog 2; DNMT3A: DNA Methyltransferase 3 Alpha; HDACs: Histone Deacetylases; MALAT1: Metastasis-Associated Lung Adenocarcinoma Transcript 1; miR-34a: MicroRNA-34a; CPT1A: Carnitine Palmitoyltransferase 1A; HADH: Hydroxyacyl-CoA Dehydrogenase; VLCAD: Very Long-Chain Acyl-CoA Dehydrogenase; ACAT2: Acetyl-CoA Acetyltransferase 2; SREBF2: Sterol Regulatory Element-Binding Transcription Factor 2; SCAP: SREBP Cleavage-Activating Protein; PGC1α: PPARG Coactivator 1 Alpha; NAMPT: Nicotinamide Phosphoribosyltransferase; SIRT1: Sirtuin 1; GPX4: Glutathione Peroxidase 4; PRDX3: Peroxiredoxin 3; SOD: Superoxide Dismutase; ROS: Reactive Oxygen Species; ATP: Adenosine Triphosphate; NAD+: Nicotinamide Adenine Dinucleotide. (Figure created by BioRender).

At the metabolic level, bCSCs exhibit pronounced metabolic duality, characterized by the ability to flexibly alternate between glycolysis and fatty acid oxidation (FAO) in response to environmental stress. The upregulation of key metabolic enzymes, such as CPT1A, not only sustains bioenergetic demands but also facilitates reactive oxygen species (ROS) clearance, thereby preserving genomic integrity [44]. Emerging preclinical evidence suggests that this transcription–metabolic coupling may critically influence immune visibility and drug responsiveness; however, the precise causal relationships underlying these effects remain to be rigorously established (Figure 3).

**Table 3:** Key stemness-associated transcription factors and their multidimensional functional integration.

Transcription Factor	Primary Regulatory Function	Metabolic Adaptation	Immune Modulation
MYC [45,46]	Activates SOX2/KLF4/NANOG-driven stemness programs; enhances self-renewal and undifferentiation	Upregulates FASN and LDHA to promote glycolysis and lipid synthesis	Maintains PD-L1 expression; stabilizes immunosuppressive signaling axes
SOX2 [47]	Maintains pluripotency; remodels chromatin accessibility; enhances stemness activity	Cooperates with EZH2 to regulate NAD^+^ metabolism and histone acetylation	Suppresses antigen presentation; reduces immune visibility of bCSCs
KLF4 [48]	Stabilizes undifferentiated states; promotes lineage reversibility	Participates in lipid metabolic reprogramming via SREBP1 regulation	Co-regulates immunotolerant environments with STAT3
FOXC1 [49]	Activates chemokines such as CXCL8 and CXCL12, promoting invasiveness	Collaborates with the cholesterol synthesis axis (SREBP2)	Drives formation of immune-exclusion zones

### 2.3. Multimodal Signaling Networks Sustaining Stemness Stability

The stability of the bCSC state is not governed by a single pathway but is instead sustained through the coordinated activity of multiple canonical signaling circuits. These include primary lineage-maintaining axes (Wnt/β-catenin and Notch), auxiliary pathways supporting metabolic fitness and quiescence (PI3K/alpha serine/threonine-protein kinase [Akt] and Hippo–YAP), and an extensive network of feedback interactions linking these modules [50,51].

Wnt/β-catenin and Notch as core stemness axes. As a central driver of stemness, Wnt signaling promotes the expression of MYC and SOX2, thereby preserving an undifferentiated lineage state. In parallel, Wnt activation induces cancer-associated fibroblasts (CAFs) to secrete microenvironmental factors such as CXCL12, further reinforcing a stemness-permissive niche [52,53,54].

Interleukin-6 (IL-6) exemplifies the context-dependent complexity of cytokine signaling in breast cancer. Beyond its established role in recruiting myeloid-derived suppressor cells (MDSCs) and regulatory T cells (Tregs) to promote immunosuppression, chronic IL-6-driven inflammation sustains long-term bCSC self-renewal through persistent STAT3 activation [55,56]. Similarly, TGF-β/Smad signaling displays marked stage specificity: while restraining proliferation in early tumorigenesis, it later facilitates epithelial–mesenchymal transition (EMT) and suppresses T-cell function, thereby reinforcing stemness maintenance and immune escape in advanced disease [57,58,59].

The Notch pathway, through Jagged1–receptor interactions, preferentially stabilizes a quiescent stem-like state and upregulates immune checkpoint molecules such as PD-L1. Regions of elevated Notch activity frequently coincide with perivascular niches, underscoring the spatial specificity of Notch-dependent stemness regulation [60,61,62].

Auxiliary pathways and network-level coupling. The PI3K/Akt pathway supports bCSC survival under stress by activating downstream effectors such as mTOR and SREBP1, thereby enhancing lipid metabolism and antioxidant capacity [63,64]. In parallel, the Hippo–YAP axis translates mechanical cues into transcriptional programs mediated by TEAD, promoting self-renewal and the expression of immune-evasive molecules [65].

**Figure 4:**
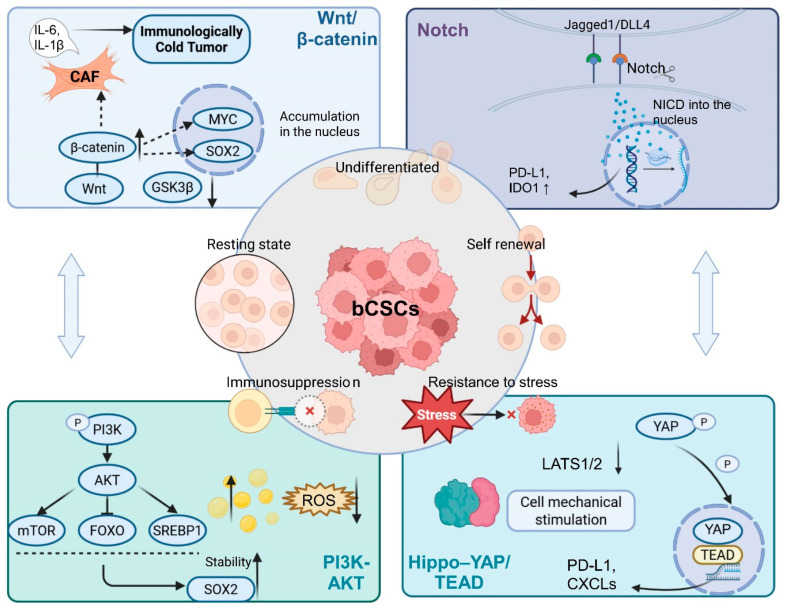
Multichannel coordinated regulation and feedback loop model governing the maintenance of bCSC states in BC. Note: bCSCs are characterized by core features including lack of differentiation, self-renewal, quiescence, immunosuppression, and stress resistance. The maintenance of these traits relies on the coordinated activation of multiple classical signaling pathways. The Wnt/β-catenin pathway promotes stemness by inhibiting GSK3β, stabilizing β-catenin, and activating MYC and SRY-box transcription factor 2 (SOX2). It also induces CAFs to secrete IL-6 and CXCL12, fostering an immunosuppressive “cold” tumor microenvironment. The Notch pathway, upon activation via Jagged1/DLL4, facilitates nuclear translocation of Notch intracellular domain (NICD), upregulates PD-L1 and IDO1, sustains quiescence, and synergizes with the YAP/TEAD axis. The PI3K/Akt pathway enhances lipid metabolism and antioxidative capacity via mTOR, FOXO, and SREBP1, thereby stabilizing SOX2 expression and promoting stress resistance. The Hippo–YAP/TEAD pathway is activated by mechanical stimuli, driving self-renewal and immune evasion through the upregulation of PD-L1 and CXCLs, and further interacts with the Notch signaling cascade. These signaling axes form interlinked positive feedback loops that construct an integrated regulatory network, endowing bCSCs with robust stability and adaptability under multifactorial stress. Abbreviations: ADAM: A Disintegrin and Metalloproteinase; LATS1/2: Large Tumor Suppressor Kinase 1/2; CXCL5: C-X-C Motif Chemokine Ligand 5. (Figure created by BioRender).

Critically, these signaling pathways are interconnected through reciprocal activation loops—for example, Wnt-mediated upregulation of PI3K signaling or cooperative regulation of YAP by Notch. Such network redundancy endows bCSCs with exceptional adaptive capacity and provides a mechanistic explanation for the frequent clinical failure of single-pathway inhibitors (Figure 4) [66,67].

### 2.4. Summary of Stemness Regulatory Networks

**Figure 5:**
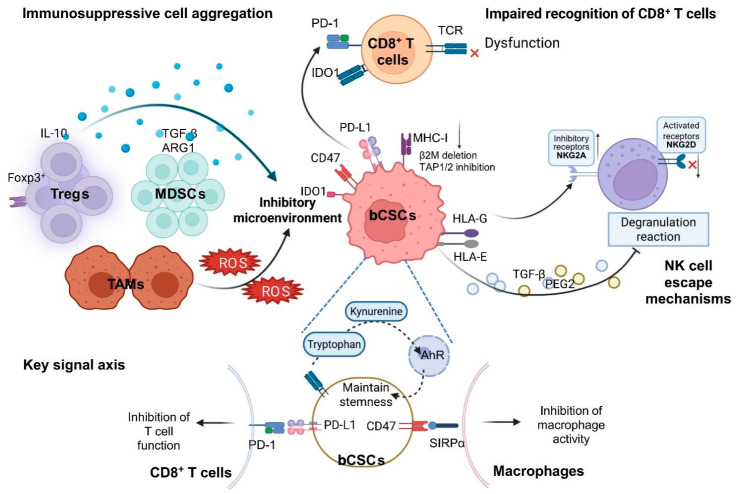
Network mechanisms of immune evasion mediated by bCSCs. Note: bCSCs evade immune surveillance and maintain their stem-like state through multiple mechanisms. First, bCSCs downregulate MHC-I, suppress TAP1/2, and lose β2M, while overexpressing PD-L1, CD47, and IDO1, collectively leading to impaired antigen presentation and functional exhaustion of CD8^+^ T cells. Second, bCSCs upregulate HLA-E/G and secrete immunosuppressive cytokines, such as TGF-β and PGE2, thereby inhibiting NK cell degranulation and promoting NK cell evasion. Simultaneously, Tregs (Foxp3^+^), MDSCs, and TAMs accumulate in bCSC-enriched regions and secrete IL-10, TGF-β, ARG1, and ROS, further establishing an immunosuppressive microenvironment. Key signaling axes involved include PD-1/PD-L1, CD47/SIRPα, and the IDO1-Kynurenine-AhR pathway, which not only attenuate immune effector cell function but also reinforce the stem-like characteristics of bCSCs through feedback mechanisms. (Figure created by BioRender).

The identification of bCSCs has progressively shifted from reliance on individual surface markers toward integrative, multi-omics-based frameworks. The maintenance of the stemness state is not an isolated process but emerges from a highly coordinated transcription–metabolic–signaling network. As illustrated in Figure 2, distinct breast cancer subtypes (e.g., TNBC versus HER2^+^ disease) exhibit subtype-specific dependencies on stemness programs. Furthermore, as shown in Figure 4 and Figure 5, core transcription factors and metabolic reprogramming are jointly reinforced by Wnt, Notch, and PI3K/Akt signaling cascades, collectively sustaining bCSC self-renewal and dynamic plasticity. Beyond stabilizing the stemness state itself, this multilayered regulatory architecture also provides the adaptive foundation that enables bCSCs to withstand immune pressure and therapeutic stress.

## 3. Reciprocal Remodeling Between Stemness and the Immune Microenvironment and Its Spatial Embedding

### 3.1. Challenges in Immune Recognition and Regulatory Barriers to Stem-like Cells

In BC, the ability of CD8^+^ T cells to recognize and eliminate tumor cells has been constrained by multiple mechanisms [68,69,70]. In specific models, researchers observed that bCSCs were not entirely immunologically invisible; under specific stimulatory conditions, they could be recognized by CD8^+^ T cells and elicited antigen-specific cytolytic responses, accompanied by reduced expression of stemness markers such as SOX2 and KLF4 [71]. This observation suggested that bCSC immune visibility was highly context-dependent and shaped by both microenvironmental cues and immune pressure [5,70].

However, in most cases, bCSCs actively suppressed antigen presentation to evade immune surveillance, through mechanisms including downregulation of MHC-I, inhibition of the TAP1/2 pathway, and loss of β2-microglobulin (β2M) [72,73,74]. In addition, bCSC subpopulations frequently expressed high levels of immunosuppressive molecules such as PD-L1, CD47, and IDO1, which interfered with T-cell and macrophage functions via the programmed cell death protein 1 (PD-1)/PD-L1 and CD47/SIRPα axes, further reducing immune clearance efficiency [75,76,77].

The capacity of natural killer (NK) cells to recognize bCSCs was also limited. Some bCSCs escaped NK-mediated cytotoxicity by upregulating inhibitory ligands such as HLA-E and HLA-G, while others secreted immunosuppressive factors such as TGF-β and PGE2 to inhibit NK cell activation [78]. Immunosuppressive cells—including Tregs, myeloid-derived suppressor cells (MDSCs), and TAMs—were frequently enriched in bCSC-dense regions, reinforcing the formation of a tolerant immune microenvironment (Figure 5) [51,79,80].

### 3.2. Signaling Communication and Remodeling of the Immune Microenvironment

In breast cancer, bCSCs are not merely passive immune escapees but act as active architects of the local microenvironment [5,51,81]. By secreting chemokines such as CCL2 and CXCL12, as well as metabolic by-products including lactate, bCSCs recruit Tregs and TAMs and establish a dual metabolic–immune suppressive barrier that disrupts dendritic cell maturation and antigen-presenting capacity [82,83].

Beyond immune cell recruitment, single-cell and spatial omics analyses have uncovered extensive ligand–receptor communication networks through which bCSCs directly condition immune cell behavior. Key signaling axes—including JAG1–NOTCH, TGF-β–TGFBR1, and CD47–SIRPα—enable bCSCs to actively induce immune exhaustion and tolerance states (Table 4) [84]. Notably, IL-6 and TGF-β play dual and context-dependent roles within this network: while mediating immunosuppression, they simultaneously function as pro-inflammatory signals that sustain stemness self-renewal via persistent STAT3 activation [85,86,87].

Importantly, next-generation computational frameworks such as CellPhoneDB and NicheNet now enable transcriptome-informed prediction of the spatial activity of these signaling axes at defined tissue coordinates. This has facilitated the systematic construction of stemness–immune pathway modules and provided a rational target landscape for combinatorial therapeutic strategies [93,94,95].

### 3.3. Trinary Coupling of Stemness, Spatial Architecture, and Immune-Cold Zones

Spatial omics studies reveal that bCSCs are not randomly distributed but are preferentially embedded within immune-shielded niches, including perivascular regions, hypoxic foci, and sub-basement membrane compartments [6,81]. These regions are frequently encapsulated by CAFs and enriched with CD206^+^ TAMs, forming a concentric architecture characterized by a stemness-enriched core, a surrounding immunosuppressive layer, and an outer zone of T-cell exhaustion [96,97]. This combined physical and functional barrier—referred to here as an immune-cold sanctuary—effectively restricts effector T-cell infiltration and constitutes a spatial basis for immunotherapy resistance [5].

Within this architectural framework, bCSCs exhibit pronounced state plasticity. Under hypoxia and immune pressure, ALDH^+^ subpopulations preferentially adopt a low-immunogenic, quiescent phenotype that minimizes immune detection, whereas CD44^+^ subpopulations located at tumor margins are more prone to transition into an invasive, activated state [98]. Further spatial functional stratification indicates that quiescent bCSCs enriched in hypoxic cores (PD-L1^+^/NR2F1^+^) and activated bCSCs localized at invasive fronts (IL-6^+^/CD44^+^) display distinct metabolic–immune signatures. This spatial heterogeneity not only governs stemness dynamics but also defines a window of plasticity for immunotherapeutic responsiveness [99,100].

**Figure 6:**
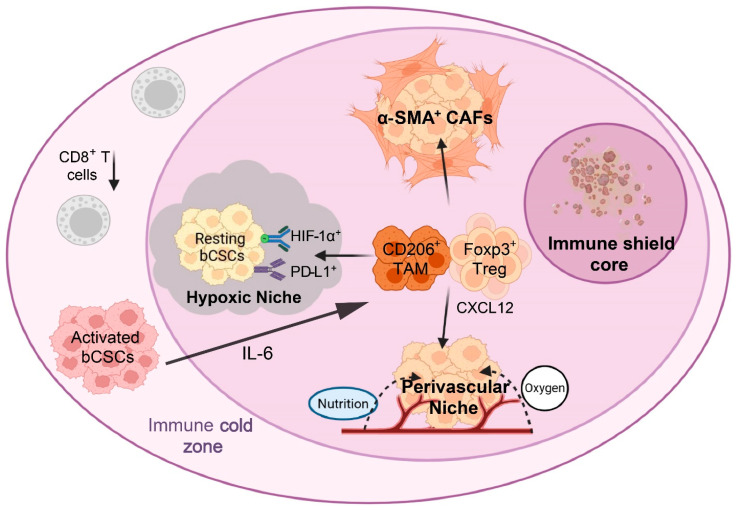
Schematic diagram of spatial niching and functional state transitions of bCSCs. Note: bCSCs actively engage in multifaceted interactions with immune and stromal cells through diverse signaling pathways, contributing to the establishment of a dynamic immunosuppressive microenvironment. On one hand, bCSCs upregulate PD-L1 and secrete factors such as IL-6 and CXCL12 to recruit TAMs and Tregs, while simultaneously suppressing CD8^+^ T-cell function. On the other hand, encapsulation by CAFs, along with hypoxic and perivascular niches, creates both physical and metabolic barriers that facilitate reversible transitions between quiescent and activated states of bCSCs. Abbreviations: bCSCs: Breast Cancer Stem Cells; TAM: Tumor-Associated Macrophages; Treg: Regulatory T-Cell; HIF-1α: Hypoxia-Inducible Factor 1-alpha; PD-L1: Programmed Death-Ligand 1; ROS: Reactive Oxygen Species. (Figure created by BioRender).

**Figure 7:**
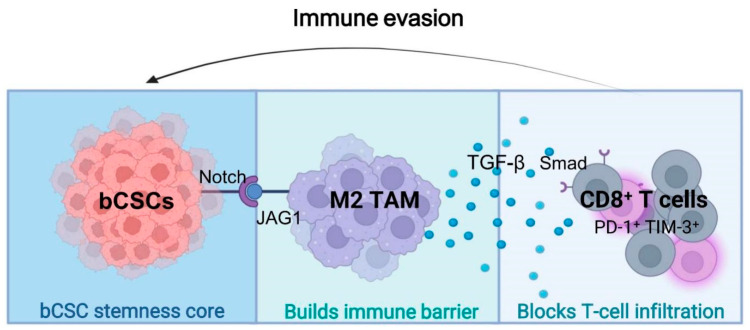
Trinary “stemness–spatial–immune” embedding structure in BC. Note: bCSCs exhibit a distinct spatial embedding pattern within the tumor microenvironment. At the core lies a bCSC-enriched zone in which Notch signaling sustains stemness and self-renewal. Surrounding this core is a middle layer composed of CD206^+^ M2-type TAMs that form an immunosuppressive barrier. These TAMs inhibit effector cell functions via the JAG1–Notch axis and TGF-β-Smad signaling pathway. The outermost layer comprises a CD8^+^ T-cell exhaustion zone, characterized by a PD-1^+^/TIM-3^+^ phenotype and sparse distribution, constituting an “immune-cold zone.” This concentric trinary structure—stemness core, suppressive barrier, and exhausted periphery—effectively impedes T-cell infiltration and cytotoxic function, thereby facilitating bCSC-driven immune evasion. Abbreviations: TAM: Tumor-Associated Macrophages; JAG1: Jagged Canonical Notch Ligand 1; TGF-β: Transforming Growth Factor Beta; PD-1: Programmed Cell Death Protein 1; TIM-3: T-cell Immunoglobulin and Mucin-domain containing-3. (Figure created by BioRender).

Collectively, this stemness–spatial–immune trinary coupling architecture (Figure 6 and Figure 7) not only shields bCSCs from immune attack but also provides a structural rationale for spatially guided precision delivery strategies [2].

### 3.4. Summary of Immune Evasion and Spatial Coupling

bCSCs are characterized not only by intrinsic defects in antigen presentation but also by their capacity to actively remodel the immune microenvironment and construct multilayered defensive barriers. As illustrated in Figure 3 and Figure 6, bCSCs recruit TAMs and Tregs through the secretion of factors such as CXCL12 and IL-6, thereby establishing an immunosuppressive niche. More critically, as shown in Figure 7, bCSCs exhibit preferential spatial embedding within immune-cold sanctuaries, giving rise to a stemness–immune–spatial trinary coupling structure. This structural barrier simultaneously impedes immune attack and provides a logical foundation for subsequent spatially targeted therapeutic delivery.

## 4. Ecological Architecture Reconstruction and Precision Intervention Guided by Multi-Omics Integration

### 4.1. Development of Stemness-Targeting Pathway Inhibitors and Combination Strategies

Substantial progress has been made in the development of inhibitors targeting core bCSC regulatory pathways (Table 5). Wnt pathway inhibitors, including LGK974 and PRI-724, have demonstrated the capacity to reverse stemness programs in organoid models. In parallel, the Notch pathway blockade—exemplified by γ-secretase inhibitors such as RO4929097—and emerging Notch4-directed antibody–drug conjugates (ADCs) are undergoing clinical or advanced preclinical evaluation, with the aim of dismantling self-renewal circuitry within bCSCs [61,101]. In addition, ALDH inhibitors (e.g., DEAB and DIMATE) and the PI3Kα inhibitor alpelisib have been shown to sensitize tumors to chemotherapy and suppress invasive phenotypes.

Despite these advances, clinical outcomes of monotherapy remain largely constrained by three interrelated factors. First, physical exclusion: spatial barriers established by bCSC niches impede effective drug penetration, limiting intratumoral target engagement [105,106]. Second, mechanistic escape: stemness-associated signaling networks exhibit high redundancy and dynamic compensatory capacity. For example, pharmacological inhibition of Wnt signaling frequently triggers feedback activation of Notch or Hedgehog pathways, a form of network-level compensation that fundamentally undermines single-axis blockade [107,108,109]. Third, the translational gap: many stemness-targeting agents operate within a narrow therapeutic window. It is critical to note that a substantial proportion of preclinical studies rely on immunodeficient models, thereby underestimating immune-related adverse events (irAEs) induced by pathway inhibition. This limitation has directly contributed to treatment discontinuation in clinical trials due to poor tolerability [108,110,111].

**Figure 8:**
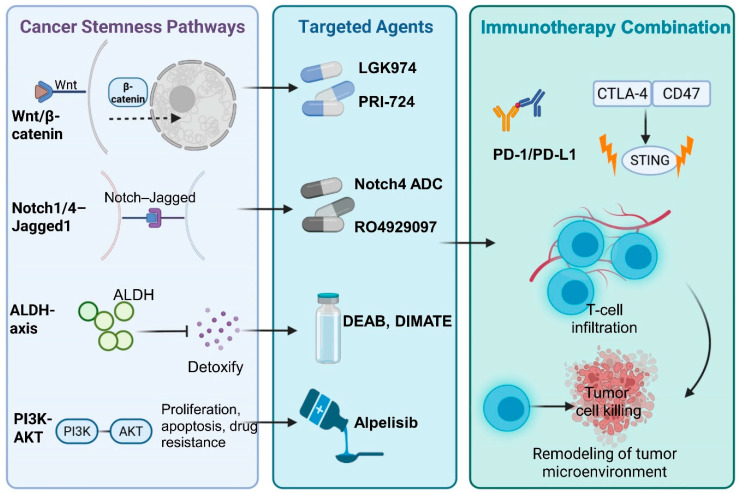
Therapeutic landscape of stemness pathway inhibition and immunotherapy combinations. Note: The stem-like state of bCSCs is sustained by multiple core signaling pathways, including Wnt/β-catenin, Notch1/4-Jagged1, ALDH, and the PI3K/Akt axis. Representative inhibitors targeting these pathways have been developed, such as the Porcupine inhibitor LGK974, the β-catenin-CBP disruptor PRI-724, Notch inhibitors RO4929097 and Notch4 ADC, ALDH inhibitors DEAB and DIMATE, and the PI3Kα inhibitor alpelisib. Several of these agents have entered clinical trials or received approval for specific indications. Studies have demonstrated that combining pathway inhibitors with immune checkpoint blockades or STING agonists can markedly enhance T-cell infiltration and cytotoxic activity, while reshaping the tumor immune microenvironment. This integrative strategy offers a promising avenue for precision targeting of bCSC-driven resistance. Abbreviations: ARG1: Arginase 1; PD-L1: Programmed Death-Ligand 1; TNF-α: Tumor Necrosis Factor Alpha; NK: Natural Killer Cell; MDSC: Myeloid-Derived Suppressor Cell; TAM: Tumor-Associated Macrophage. (Figure created by BioRender).

In light of these constraints, future therapeutic strategies must shift from single-axis suppression toward network-oriented combinatorial intervention. In particular, nanotechnology-based delivery systems offer a promising means to refine therapeutic windows by enhancing spatial precision and reducing systemic toxicity [112,113,114]. Moreover, accumulating evidence indicates robust synergy between stemness pathway inhibitors and PD-1 blockade. This integrated strategy—combining stemness eradication with immune reactivation—represents a compelling route to overcoming therapeutic resistance and reshaping the bCSC ecological niche (Figure 8) [115,116,117].

### 4.2. Disrupting Spatial Barriers and Coordinated Immune Activation

To overcome the immune-cold zones and multilayered escape barriers established by bCSCs, combinatorial strategies that integrate immune-activating pathways with stemness pathway inhibition have emerged as a promising direction. Two complementary approaches are central to this paradigm.

(1) Immune activation. STING agonists, such as ADU-S100, activate the cGAS–STING axis and induce the production of inflammatory chemokines, thereby promoting T-cell recruitment into previously immune-excluded regions. In parallel, blockade of the CD47 “don’t eat me” signal using anti-CD47 antibodies (e.g., magrolimab) restores macrophage-mediated phagocytosis within stemness-enriched niches, enhancing innate immune clearance [118,119].

(2) Spatially guided delivery. Advances in spatial omics now enable the identification of PD-L1^+^/CD8^−^ hotspots using platforms such as Visium. This information can be leveraged to design targeted nanodelivery systems—including liposomes and hydrogel microspheres—that enable localized co-release of Wnt/Notch inhibitors and immune activators within defined tumor regions [120].

**Figure 9:**
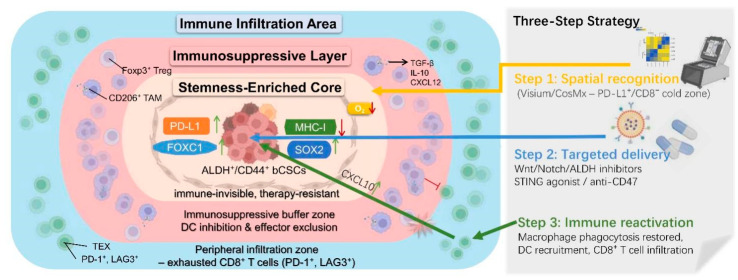
Mechanistic model of combination therapy targeting the spatial structure of the stemness-driven immune-cold niche. Note: bCSCs frequently establish a three-layered spatial architecture of the “stemness–immune-cold zone”, consisting of a central core, an immunosuppressive barrier, and a peripheral infiltration zone. Within this structure, only a sparse population of CD8^+^ T cells is present, primarily exhibiting an exhausted phenotype and failing to penetrate the central core. To overcome this barrier, a three-step therapeutic strategy is proposed: Step 1: Utilize spatial transcriptomics platforms to identify PD-L1^+^/CD8^−^ “cold zones”; Step 2: Employ targeted delivery of Wnt/Notch/ALDH inhibitors, STING agonists, or anti-CD47 antibodies to disrupt the stemness core and the surrounding immune shield; Step 3: Reconstruct antitumor immune responses, ultimately converting the cold zone into a “hot zone” and inducing a durable cytotoxic immune response. Abbreviation: CD3: Cluster of Differentiation 3. (Figure created by BioRender).

This integrated strategy—combining target-zone localization, pathway disruption, and immune reactivation—aims to simultaneously eliminate the stemness core and reconstruct a permissive immune ecosystem. Superior synergistic efficacy compared with monotherapy has been demonstrated in organoid and humanized models, supporting its potential to dismantle immune-cold sanctuaries and provide more durable therapeutic benefit in treatment-refractory breast cancer (Figure 9) [121].

### 4.3. Multimodal Model Validation and AI-Assisted Decision Systems

To balance mechanistic depth with translational relevance and overcome the limitations of single-model systems, bCSC research is increasingly adopting a multimodal framework built upon hierarchical model validation and AI-assisted decision-making.

#### 4.3.1. Hierarchical Validation Pipeline

Distinct experimental questions necessitate a structured hierarchical validation pipeline:

- Level I (mechanistic screening): Patient-derived organoids (PDOs) enable high-throughput drug screening with preserved tissue architecture, making them well suited for evaluating Wnt- and Notch-targeting agents [122].
- Level II (subtype adaptation): PDX models are used to validate prioritized compounds in specific subtypes (e.g., TNBC), allowing assessment of intratumoral penetration and antitumor efficacy within an intact in situ microenvironment [123].
- Level III (immune interaction): Humanized mouse models reconstruct functional human immunity and enable precise evaluation of T-cell activation elicited by combinations of PD-1 blockade and stemness inhibitors—an interaction that conventional PDX models cannot capture [124].

**Figure 10:**
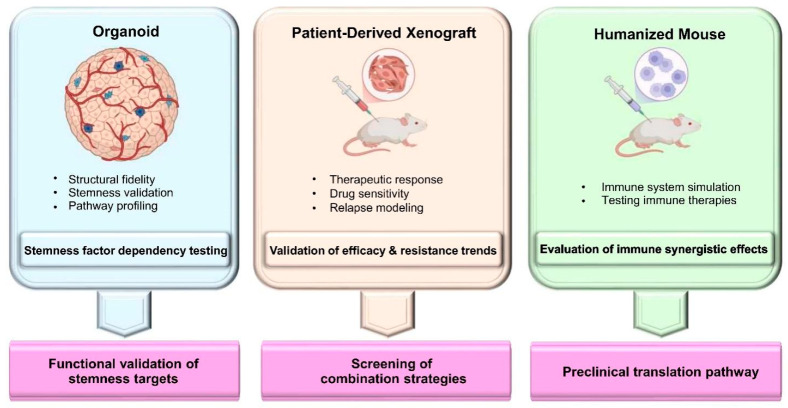
Validation systems based on organoids, PDX, and humanized models. Note: Three pivotal experimental models support the validation of bCSC targets and combination treatment strategies in BC: (i) organoids preserve structural fidelity and enable in vitro testing of stemness-related dependencies and pathway/transcriptome-specific analyses; (ii) PDX models are used to assess drug sensitivity and therapeutic responses, replicate tumor relapse and metastasis, and uncover drug resistance trajectories; (iii) humanized mouse models reconstruct the human immune system, allowing for evaluation of the synergistic effects of immune combination therapies. Together, these platforms support a translational research pipeline from functional validation of stemness regulators to combination strategy screening and clinical implementation. (Figure created by BioRender).

Together, this integrated pipeline (Figure 10) ensures end-to-end validation from molecular mechanisms to immune responses.

#### 4.3.2. Integration of Spatial Omics and AI

Spatial omics platforms (e.g., Visium) have revealed the ecological organization of bCSC niches but remain constrained by the trade-off between resolution and coverage. Two complementary strategies have been proposed to overcome this limitation:

(i) Algorithmic resolution enhancement, whereby deconvolution frameworks such as Cell2location integrate scRNA-seq data to enable virtual “super-resolution” inference of cell–cell communication; and (ii) throughput expansion, applying spatial technologies to tissue microarrays (TMAs) to increase cohort-scale applicability [125].

**Figure 11:**
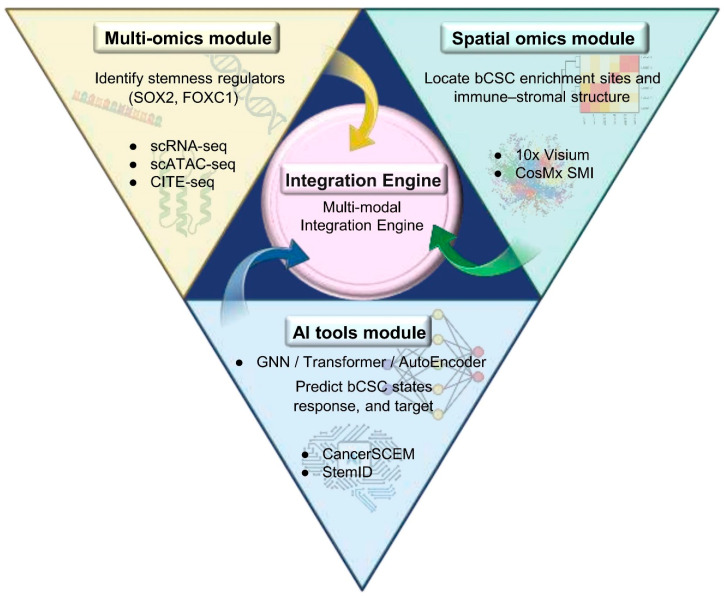
Integrated triaxial model of multi-omics, spatial omics, and AI technologies. Note: This model illustrates the synergistic interplay among three core technological domains: (i) Multi-Omics Module: Utilizes scRNA-seq, scATAC-seq, and CITE-seq to identify lineage-specific stemness regulators (e.g., SOX2, FOXC1) and to characterize transcriptional and epigenetic features of bCSCs; (ii) Spatial Omics Module: Employs platforms such as 10x Visium and CosMx SMI to map bCSC-enriched regions and delineate their spatial associations with immune and stromal components; (iii) AI Tools Module: Leverages algorithms including GNNs, Transformers, and AutoEncoders to predict bCSC states, infer treatment responses, and recommend combinatorial therapeutic targets. Representative platforms include CancerSCEM and StemID. These modules converge through an integrative engine to advance closed-loop mechanistic dissection of bCSCs and to optimize precision-tailored therapeutic strategies. Abbreviations: scRNA-seq: Single-Cell RNA Sequencing; GNN: Graph Neural Network; SOX2: SRY-Box Transcription Factor 2; FOXC1: Forkhead Box C1. (Figure created by BioRender).

When coupled with AI tools such as graph neural networks (GNNs), these approaches enable high-fidelity prediction of bCSC–immune communication networks and spatial interaction patterns (Figure 11).

#### 4.3.3. AI-Driven Clinical Decision Systems

**Figure 12:**
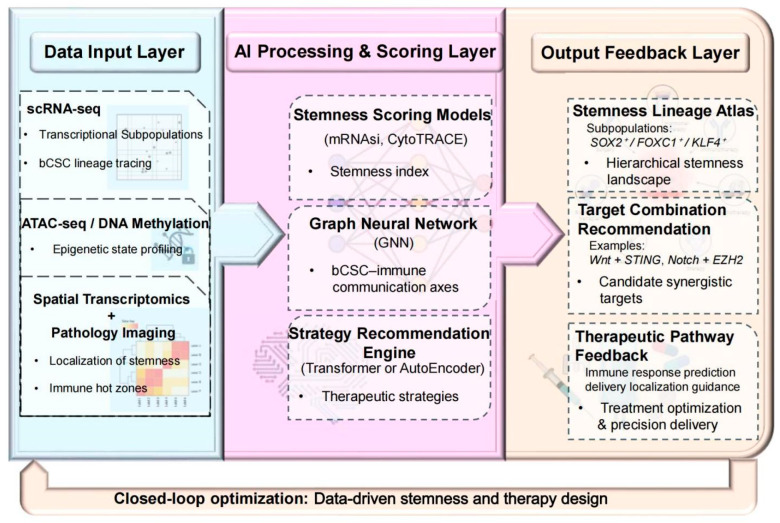
Closed-loop system for stemness identification, target recommendation, and therapeutic feedback based on multi-omics and AI. Note: This framework consists of three functional layers: (i) Data Input Layer: Incorporates scRNA-seq for transcriptional subtype classification, ATAC-seq/DNA methylation profiling for epigenetic state characterization, and spatial transcriptomics combined with histopathology to map stemness–immune hot/cold regions; (ii) AI Processing and Scoring Layer: Utilizes stemness scoring algorithms (e.g., mRNAsi, CytoTRACE) and GNNs to identify bCSC–immune communication axes and generate combination therapy recommendations via a strategy engine; (iii) Output and Feedback Layer: Produces lineage-specific stemness maps, recommends synergistic target pairs (e.g., Wnt + STING, Notch + EZH2), and provides treatment response predictions and guidance for precision drug delivery. This system enables data-driven optimization of bCSC identification and individualized therapeutic design. Abbreviations: scRNA-seq: Single-Cell RNA Sequencing; ATAC-seq: Assay for Transposase-Accessible Chromatin with High-Throughput Sequencing. (Figure created by BioRender).

To establish clinically reliable individualized treatment systems, several key dimensions require optimization (Figure 12):

- Transfer learning: Leveraging pan-cancer datasets for pretraining to mitigate sample scarcity in rare subtypes;
- Causal inference: Integrating CRISPR perturbation data to distinguish stemness-associated features from true stemness drivers;
- Explainable AI (XAI): Employing SHAP values or attention heatmaps to visualize model decisions, annotate high-risk microdomains, and incorporate uncertainty quantification to flag low-confidence predictions.

Ultimately, standardization and clinical adoption will depend on the establishment of an internationally shared bCSC spatial atlas reference dataset and the execution of multicenter prospective clinical trials to validate predictive performance and clinical utility [126].

### 4.4. Summary of Therapeutic Strategies

**Figure 13:**
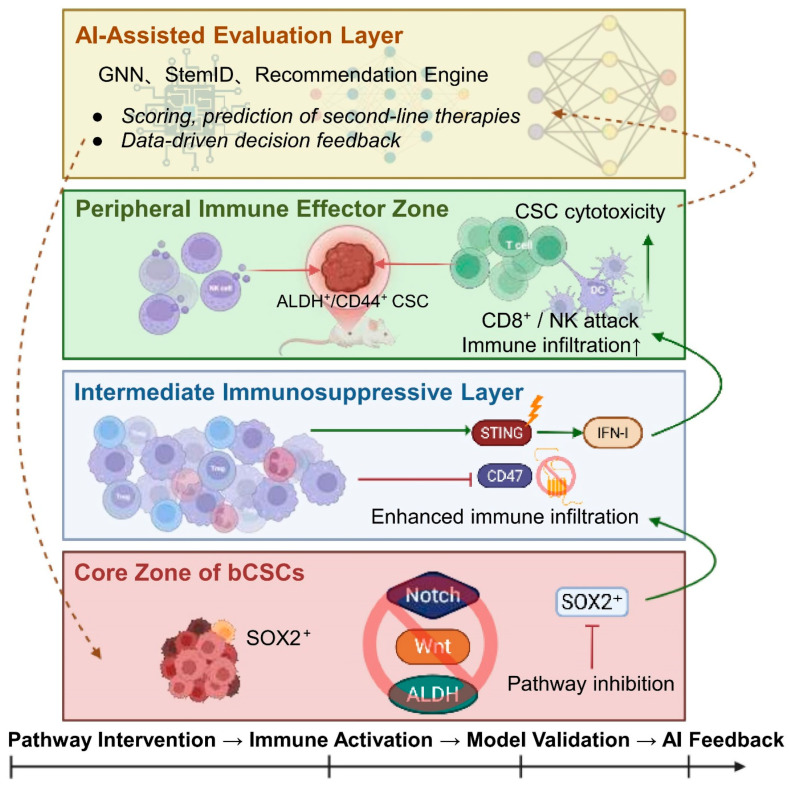
Multilayered strategic framework integrating bCSC-targeted therapy, immune modulation, and AI-driven systems. Note: The therapeutic framework for targeting bCSCs is structured vertically into four distinct layers: (i) Core Stemness Zone: Characterized by SOX2^+^ bCSCs sustained by Wnt, Notch, and ALDH signaling pathways; (ii) Intermediate Immunosuppressive Layer: Composed of Tregs, MDSCs, and TAMs, which establish immune barriers via CD47 and STING signaling axes; (iii) Peripheral Immune Effector Zone: Encompasses infiltrating CD8^+^ T cells and NK cells mediating cytotoxic responses; (iv) AI-Assisted Evaluation Layer: Employs algorithms such as GNNs and StemID to perform stemness scoring, therapeutic strategy recommendation, and efficacy prediction. Horizontally, the workflow forms a closed-loop from pathway intervention → immune activation → model validation → AI feedback. Abbreviations: AI: Artificial Intelligence; GNN: Graph Neural Network; STING: Stimulator of Interferon Genes. (Figure created by BioRender).

Given the multilayered regulation and spatial shielding of bCSCs, single-modality therapies are unlikely to achieve durable control. As illustrated in Figure 8, combinations of stemness pathway inhibitors and immunotherapies exhibit clear synergistic potential. As shown in Figure 9, spatial barrier-disrupting strategies targeting immune-cold zones aim to restore immune infiltration. To validate these approaches, an integrated framework combining organoids, PDX models, and AI-driven multi-omics prediction systems (Figure 10, Figure 11, Figure 12 and Figure 13) establishes a closed-loop pathway from mechanistic insight to clinical decision-making.

## 5. Summary

Despite rapid advances in the study of bCSCs, several fundamental challenges remain at the level of mechanistic understanding.

First, the challenge of dynamic state definition. bCSCs are no longer best described as a static, rare subpopulation; instead, they represent a reversible and highly plastic functional state capable of multilineage transitions. Their dynamic adaptability across metabolic, immune, and transcriptional dimensions precludes comprehensive characterization by any single marker or experimental model. This intrinsic state fluidity constitutes a central obstacle in explaining therapeutic resistance and disease recurrence.

Second, the challenge of heterogeneity in identification systems. Widely used identification schemes (e.g., CD44^+^/CD24^−^, ALDH^+^) display substantial variability in stability across breast cancer subtypes. This limitation becomes particularly evident in spatial omics contexts, where discordance frequently arises between phenotype and function—manifesting as phenotypically positive but functionally inactive cells, or functionally stem-like cells with ambiguous phenotypes. Moreover, systematic investigations delineating subtype-specific differences in bCSC phenotypes, signaling dependencies, and druggable targets remain scarce, impeding the development of precision-aligned targeting strategies.

Third, the challenge of causal validation. Direct causal links between bCSCs and clinical treatment responses—such as therapeutic resistance or immune non-responsiveness—have yet to be firmly established. Most existing studies rely predominantly on correlative evidence and lack robust functional systems for causal interrogation, thereby constraining mechanistic resolution and slowing the translation of stemness insights into targeted drug development. In parallel, current spatial transcriptomics platforms (e.g., 10× Visium) remain limited by the trade-off between resolution and coverage, restricting comprehensive decoding of fine-grained microenvironmental networks and underscoring the need for higher-precision technological breakthroughs.

## 6. Outlook: Toward Spatially Precise Intervention and Digitally Integrated Stemness Medicine

Over the next 3–5 years, progress in bCSC research will depend on addressing three interrelated priorities.

### 6.1. From Correlation to Causation: Mechanistic Validation

Future experimental strategies must move decisively beyond associative observations to establish causality. On one hand, high-resolution lineage-tracing approaches—such as CRISPR barcoding—should be employed to track bCSC clonal dynamics under therapeutic pressure at the single-cell level, directly testing whether bCSCs function as ancestral founders of recurrent disease. On the other hand, the development of spatially resolved optogenetic or photothermal perturbation systems will enable precise spatiotemporal elimination or functional modulation of bCSCs within defined ecological niches (e.g., immune-cold zones) under in vivo imaging guidance. Demonstration of concomitant microenvironmental remodeling and reversal of resistance following such interventions would provide compelling in vivo causal evidence for the driver role of bCSCs.

### 6.2. From Retrospective to Prospective: Clinical Validation

To enable standardized clinical deployment of AI-assisted systems, an internationally shared bCSC spatial atlas benchmark dataset must be established, accompanied by multicenter prospective clinical trials. By comparing outcomes between AI-guided treatment arms and standard-of-care cohorts in real-world heterogeneous populations—and by subjecting algorithmic frameworks to open-source auditing—both clinical efficacy and ethical transparency can be rigorously validated. Such efforts are essential for defining evidence-based performance standards and building clinical trust.

### 6.3. From Single-Point Intervention to a Digital Closed Loop

The ultimate objective is the construction of a spatial–mechanistic–therapeutic closed-loop system that is capable of identification, intervention, tracking, and adaptive feedback:

- Identification layer: Multi-omics and spatial data are used to map bCSC distribution and state;
- Assessment layer: AI and machine learning models generate stemness scores and risk predictions;
- Intervention layer: Regionally targeted delivery and immune reprogramming strategies disrupt protective barriers and amplify immune effector functions;
- Feedback layer: Imaging and biomarker-based monitoring informs therapeutic efficacy, enabling iterative target reprioritization and optimization of combination regimens.

**Figure 14:**
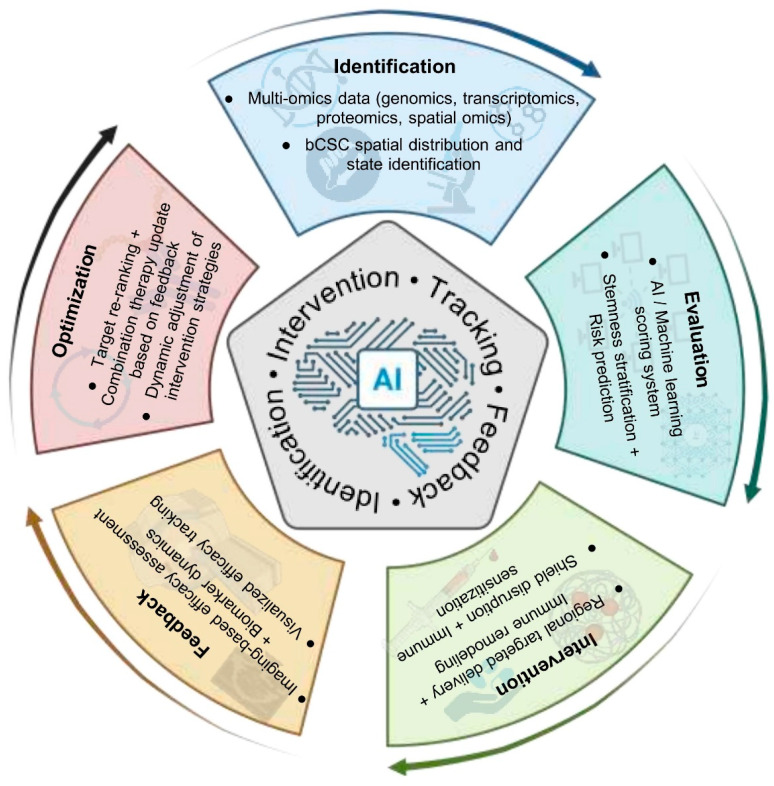
Closed-loop decision-making platform for precision intervention of stemness. Note: This AI-centric framework comprises a five-stage closed-loop system encompassing identification, evaluation, intervention, feedback, and optimization. Initially, multi-omics and spatial omics data are integrated to map the distribution and functional states of bCSCs. Next, AI and machine learning models are applied to generate stemness scores and predict associated risks. Targeted regional delivery combined with immune remodeling strategies is then employed to disrupt bCSC-associated barriers and enhance antitumor immune responses. Therapeutic efficacy is monitored through imaging and biomarker-based feedback. Finally, the feedback is used to reprioritize targets and update combination regimens, enabling continuous refinement of treatment strategies. This closed-loop platform is designed to achieve a digitally guided stemness intervention pathway that is identifiable, actionable, traceable, and responsive. (Figure created by BioRender).

Collectively, these advances signal the transition of bCSC research into a new era defined by the convergence of spatial biology, mechanistic rigor, and intelligent systems. This integrative trajectory is poised to propel breast cancer treatment toward a genuinely digitally enabled, spatially precise precision medicine paradigm (Figure 14).

## Figures and Tables

**Table 1:** Combinatorial biomarkers and contextual applicability for identifying bCSCs.

Biomarker Combination	Functional Significance	Advantages	Subtype Compatibility	Detection Method
CD44^+^/CD24^−^ [31]	Migratory stem-like phenotype; promotes EMT and invasiveness	Phenotypically stable; broadly applicable	TNBC, basal-like	FACS, IHC
ALDH^+^ [7]	Quiescent stemness phenotype with high drug resistance	Co-localized with immune-cold zones; highly chemoresistant	TNBC, high-grade HER2^+^	ALDEFLUOR assay kit
EpCAM^+^/CD49f^+^ [32]	Epithelial–basal stem-like phenotype with high lineage plasticity	Indicates reversibility of stemness trajectory	Luminal A, HER2^+^	Dual-channel FACS or CyTOF
mRNAsi Score (AI-Based Prediction) [33]	Quantifies stemness level via transcriptomic modeling	Antibody-independent; suitable for large-scale screening	Multiple subtypes	Bulk RNA-seq + machine learning

**Table 2:** Key differences and similarities between breast cancer stem-like cells (bCSCs) and non-stem breast cancer cells (non-bCSCs).

Feature	Breast Cancer Stem-Like Cells (bCSCs)	Non-Stem Breast Cancer Cells (Non-bCSCs)	Significance
Self-renewal	High (Unlimited potential)	Low (Limited potential)	Drives tumor initiation & recurrence
Differentiation	Multipotent (Can differentiate into non-bCSCs)	Restricted (Terminally differentiated)	Generating cellular heterogeneity
Surface Markers	CD44^+^/CD24^−^, ALDH1^+^, EpCAM^+^	CD44^−^/CD24^+^, ALDH1^−^	Targets for identification & isolation
Chemotherapy	Resistant (Quiescent state, ABC transporters high)	Sensitive (Rapidly proliferating)	Cause of therapeutic failure
Metabolism	Plastic (High OXPHOS/FAO dependency)	Glycolytic (Warburg effect dominant)	Metabolic vulnerability for targeting
Immune Profile	Immune-evasive (Low MHC-I, High PD-L1/CD47)	Immunogenic (Higher antigen presentation)	Escape from immune surveillance
Tumorigenicity	High (Can form tumors at low cell numbers)	Low (Requires high cell numbers)	Gold standard for functional validation
Plasticity	High (Can interconvert between states)	Low (Stable phenotype)	Adaptation to microenvironmental stress

**Table 4:** Key ligand–receptor signaling axes between bCSCs and immune cells.

Ligands Derived from bCSCs	Receptor on Target Immune Cells	Functional Outcome	Associated Pathway	Spatial Localization
JAG1 [88]	NOTCH1 (TAMs)	Activates TAM quiescence; promotes immune tolerance	Notch pathway	Perivascular niche
CD47 [89]	SIRPα (macrophages)	Inhibits phagocytosis; forms “don’t eat me” signal	SIRPα–Shp axis	Core bCSC zone
CXCL12 [90]	CXCR4 (Tregs)	Recruits regulatory T cells; induces local immune suppression	Chemokine axis	CAF-encapsulated region
Galectin-9 [91]	Tim-3 (CD8^+^ T cells)	Induces T-cell exhaustion	Immune checkpoint axis	CD8^+^ T-cell-low zones
TGF-β [92]	TGFBR1 (DCs, T cells)	Suppresses antigen presentation; fosters cold immune niches	TGFβ–Smad pathway	bCSC–TAM embedded region

**Table 5:** Targeted therapy strategies for bCSCs: Integration of pathway inhibitors and immune synergistic agents.

Targeted Pathway	Core Function	Representative Agents	Clinical Status/Model Validation
Wnt/β-catenin [102]	Maintains stem cell lineage; suppresses antigen presentation	LGK974	Validated in human HNSCC models
Notch4 [103]	Sustains homeostasis; establishes quiescence	Caerulein	Confirmed in invasive PDAC mouse models
ALDH [27]	Supports quiescent stemness and drug efflux	KK-LC-1	Validated in ALDH^+^ stem cell populations
PI3K/Akt [104]	Enhances antioxidant capacity; stabilizes stemness	RLY-2608	Demonstrated mutant selectivity in patients

## Data Availability

The original contributions presented in this study are included in the article. Further inquiries can be directed to the corresponding author.

## Abbreviations

ABC Transporter	ATP-Binding Cassette Transporter
ADC	Antibody–Drug Conjugate
AI	Artificial Intelligence
ALDH	Aldehyde Dehydrogenase
ATAC-seq	Assay for Transposase-Accessible Chromatin with High-Throughput Sequencing
BC	Breast Cancer
bCSCs	Breast Cancer Stem-like Cells
CAFs	Cancer-Associated Fibroblasts
CCL2	C-C Motif Chemokine Ligand 2
CCL5	C-C Motif Chemokine Ligand 5
CD	Cluster of Differentiation (e.g., CD44, CD24, CD47, CD206)
ceRNA	Competing Endogenous RNA
CITE-seq	Cellular Indexing of Transcriptomes and Epitopes by Sequencing
CPT1A	Carnitine Palmitoyltransferase 1A
CSCs	Cancer Stem Cells
CXCL12	C-X-C Motif Chemokine Ligand 12
CXCR4	C-X-C Motif Chemokine Receptor 4
DNMT3A	DNA Methyltransferase 3 Alpha
EMT	Epithelial–Mesenchymal Transition
EpCAM	Epithelial Cell Adhesion Molecule
EZH2	Enhancer of Zeste Homolog 2
FACS	Fluorescence-Activated Cell Sorting
FAO	Fatty Acid Oxidation
FOXC1	Forkhead Box C1
GNN	Graph Neural Network
GSK3β	Glycogen Synthase Kinase 3 Beta
HADH	Hydroxyacyl-CoA Dehydrogenase
HDACs	Histone Deacetylases
HER2+	Human Epidermal Growth Factor Receptor 2-Positive
HLA	Human Leukocyte Antigen
IDO1	Indoleamine 2,3-Dioxygenase 1
IHC	Immunohistochemistry
IL-6	Interleukin-6
irAEs	Immune-related Adverse Events
KLF4	Krüppel-Like Factor 4
MDSCs	Myeloid-Derived Suppressor Cells
MHC-I	Major Histocompatibility Complex Class I
NAD+	Nicotinamide Adenine Dinucleotide
NAMPT	Nicotinamide Phosphoribosyltransferase
NICD	Notch Intracellular Domain
NK cells	Natural Killer cells
OCT4	Octamer-Binding Transcription Factor 4
PD-1	Programmed Cell Death Protein 1
PD-L1	Programmed Death-Ligand 1
PDX	Patient-Derived Xenograft
PDOs	Patient-Derived Organoids
PGC1α	PPARG Coactivator 1 Alpha
PI3K	Phosphoinositide 3-Kinase
PRDX3	Peroxiredoxin 3
ROS	Reactive Oxygen Species
scRNA-seq	Single-Cell RNA Sequencing
SCAP	SREBP Cleavage-Activating Protein
SHAP	SHapley Additive exPlanations
SIRPα	Signal Regulatory Protein Alpha
SIRT1	Sirtuin 1
SOD	Superoxide Dismutase
SOX2	SRY-Box Transcription Factor 2
SREBF2	Sterol Regulatory Element-Binding Transcription Factor 2
SREBP1	Sterol Regulatory Element-Binding Protein 1
STING	Stimulator of Interferon Genes
TAMs	Tumor-Associated Macrophages
TAP	Transporter Associated with Antigen Processing
TEAD	TEA Domain Transcription Factor
TF	Transcription Factor
TGF-β	Transforming Growth Factor Beta
TIM-3	T-cell Immunoglobulin and Mucin-domain containing-3
TMA	Tissue Microarray
TNBC	Triple-Negative Breast Cancer
Tregs	Regulatory T Cells
VLCAD	Very Long-Chain Acyl-CoA Dehydrogenase
XAI	Explainable Artificial Intelligence
YAP	Yes-Associated Protein
β2M	Beta-2-Microglobulin
